# Mitochondria Associated MicroRNA Expression Profiling of Heart Failure

**DOI:** 10.1155/2017/4042509

**Published:** 2017-09-24

**Authors:** Xiaoxia Wang, Chun Song, Xiao Zhou, Xiaorui Han, Jun Li, Zengwu Wang, Haibao Shang, Yuli Liu, Huiqing Cao

**Affiliations:** ^1^Institute of Molecular Medicine, Peking University, Beijing, China; ^2^Laboratory Animal Center, Peking University, Beijing, China; ^3^Division of Prevention and Community Health, National Center for Cardiovascular Disease, Fuwai Hospital, Peking Union Medical College, Chinese Academy of Medical Sciences, Beijing, China

## Abstract

Heart failure (HF) is associated with mitochondrial dysfunction and energy metabolism impairment. MicroRNAs are implicated in the development of heart failure. However, the mitochondria enriched microRNA during heart failure remains elusive. Here, we generated a pressure overload-induced early and late stage heart failure model at 4 weeks and 8 weeks following transverse aortic constriction (TAC) in mice. We found that expression of mitochondrion protein COX4 was highly enriched in isolated mitochondria from cardiac tissues while GAPDH could hardly be detected. Furthermore, small RNA sequencing for mitochondria RNAs from failing hearts was performed. It was found that 69 microRNAs were upregulated and 2 were downregulated in early heart failure, while 16 microRNAs were upregulated and 6 were downregulated in late heart failure. 15 microRNA candidates were measured in both mitochondria and total cardiac tissues of heart failure by real-time PCR. MiR-696, miR-532, miR-690, and miR-345-3p were enriched in mitochondria from the failing heart at early stage. Bioinformatics analysis showed that mitochondria enriched microRNAs in HF were associated with energy metabolism and oxidative stress pathway. For the first time, we demonstrated microRNAs were enriched in mitochondria during heart failure, which established a link between microRNA and mitochondrion in heart failure.

## 1. Introduction

Heart failure (HF) is a growing public health concern worldwide. It is the common end stage of diverse heart diseases and has been the leading cause of morbidity, mortality, and hospitalization in the elderly people. Despite significant advances in the etiology and therapeutic strategies were made in the past decades, new targets for better prognosis and treatment in HF are still urgently needed.

The constantly beating heart demands great energy, which is mainly provided by the large amount of mitochondria within each individual cell. Mitochondria represent 30% of the total volume of the cardiomyocytes and act as critical integrators of energy production, reactive oxygen species (ROS) generation, cell death, and signaling transduction [1]. A key characteristic feature in the development of heart failure is the imbalance between ATP demand and production. Cardiac mitochondrial dysfunction is associated with heart failure of diverse etiologies [2].

MicroRNAs are 20–22 nt small noncoding RNA molecules that regulate gene expression via posttranscription and/or translational repression [3]. Studies have demonstrated the involvement of microRNAs in several processes related to HF, such as oxidative stress, energy metabolism, cell survival, and apoptosis in the pathogenesis [4–8]. Differential circulating microRNA profiles were determined in HF cohorts, suggesting the diagnostic utility of microRNAs [9]. Several key microRNAs such as miR-208a, miR-221, and miR-340-5p were identified in failing hearts. Alternations in these microRNAs contributed to the occurrence and progression of heart failure [6–8].

MicroRNAs are mainly located in cytosol or nucleus. Recent studies suggested that microRNAs contain sequence elements that affect their subcellular localization [10]. Moreover, Ago2 has been implicated as a carrier to transport microRNAs between mitochondria and cytosol [11]. A number of studies have shown that microRNAs can translocate into the mitochondria at either pre-RISC or mature-RISC conformation [12], indicating a special impact of these microRNAs on mitochondrial function. For example, miR-1 stimulated mitochondrial genome-encoded transcripts by Ago2-mediated translocation into the mitochondria during muscle differentiation [13]. Similarly, miR-181c and miR-378 regulated mitochondria targets in cardiomyocytes, leading to alteration in electron transport chain, fatty acid metabolism, and apoptosis under metabolic stress [14–16]. A new term “MitomiR” was created for the microRNAs localized in mitochondria to regulate gene functions [11, 17]. These microRNAs regulate specific pathways contributing to energy metabolism at the mitochondrial level [18, 19].

Given the importance of mitochondria and microRNAs in heart diseases, we hypothesized that mitochondria associated microRNAs may represent a different regulatory layer of HF. However, the expression profiling of mitochondria associated microRNAs in heart failure is still unknown. In the current study, we isolated cardiac mitochondrion RNA from a heart failure mice model and performed small RNA sequencing. For the first time, we demonstrated that the expression profiling of mitochondria associated microRNAs in HF. miR-696, miR-532, miR-690, and miR-345-3p were found to be enriched in mitochondria of failing hearts. Bioinformatics analysis indicated that these mitochondria enriched microRNAs in HF were associated with energy metabolism and oxidative stress pathway.

## 2. Materials and Methods

### 2.1. Animal Model

Animal procedures in the present study conform to the requirements of the Animal Welfare Act. The Institutional Animal Care and Use Committee (IACUC) at the Peking University approved the protocols. Wild-type C57BL/6 J mice (10~12-week-old male, 25 ± 5 g body weight) were obtained from* Weitonglihua* experiment limited company (Beijing, China). Heart failure was induced by pressure overload after transverse aortic constriction (TAC) surgery as previously described [20]. In brief, mice were anesthetized with ketamine (25 mg/kg) and were mechanically ventilated. Aortic constriction was created via a center thoracotomy, and ligation of the transverse thoracic aorta was performed with a 28-gauge needle using a 7-0 braided polyester suture. After ligation, the needle was removed, the chest and skin were closed, and the mice were allowed to recover. Sham-operated mice underwent the same procedure without constriction. Mouse hearts were excised at 4 or 8 weeks after TAC.

### 2.2. Echocardiography and Hemodynamic Analysis

At 4 weeks or at 8 weeks after surgery, transthoracic 2D and M-mode echocardiography were performed from the long-axis view of the heart at the level of the papillary muscle using a Vevo 770 echocardiography system (Visual Sonics, Canada) with a 30 MHz linear array transducer. The mice were anesthetized with isoflurane (2%). Left ventricular end-diastolic internal diameter (LVIDd) and left ventricular end-systolic internal diameter (LVIDs) were measured in M-mode. Fraction shortening (FS) and ejection fraction (EF) were calculated. Measurements were averaged from five separate cardiac cycles.

### 2.3. Mitochondria Isolation and Purification

All procedures were performed on wet ice. Mouse hearts were rapidly minced in ice cold MSE buffer containing 220 mM mannitol, 70 mM sucrose, 1 mM EDTA, 5 mM Tris HCL (pH 7.5), and 0.5 mg/ml bovine serum albumin (BSA). Heart tissues were homogenized in MSE buffer, using a pestle by stroking the minced samples. Homogenates were then centrifuged at 800 ×g for 5 min. Supernatants were further centrifuged twice at 4,000 ×g for 5 min at 4°C, and crude pellets were resuspended and submitted to further purification by Percoll gradient fractionation in 0.3 M sucrose and 10 mM MOPS/KOH (pH 7.2). After 45 min ultracentrifugation at 70,000 ×g (Beckman SW 40 rotor, Beckman Coulter), intact mitochondria were isolated from the interphase. The mitochondria were resuspended and centrifuged again for further purification. RNase treatment was performed to remove cytosolic RNA contamination on the surface of mitochondrial membranes.

### 2.4. RNA Isolation and Sequencing

Total RNA was isolated from heart tissues or mitochondria using Trizol reagent (Invitrogen) and treated with RNase-free DNase I for 1 h. The quantity and quality of obtained RNA were measured with spectrophotometer, Nanodrop ND-1000. We combined RNAs from 12 different individuals with equal molar amount into a single pooled sample for each group. Small RNA library preparation and sequencing were performed by Beijing Genomics Institute (BGI, Beijing, China), following Illumina's protocols for HiSeq2000 single-end sequencing (1 × 50 bp).

### 2.5. Quantitative RT-PCR

For microRNA quantification, stem-loop reverse transcription followed by qPCR analysis was used. 500 ng total RNA was reverse transcribed using specific stem-loop primers (purchased from RiboBio Co., Ltd) and SYBR Green-based qPCR was carried out using specific forward primer and universal reverse primer (RiboBio Co., Ltd), U6 was used as an internal control. Primers for U6 are 5′CTCGCTTCGGCAGCACA 3′ (F), U6 5′AACGCTTCACGAATTTGCGT3′ (R). For mRNA qRT-PCR, 500 ng DNase I digested total RNA was reverse transcribed using oligo (dT) primer (TransGen Biotech) and gene specific primers were used for SYBR Green (Invitrogen) based qPCR. Primers for *α*-MHC are 5′CGACTACGCCTTCGTCTCTC-3′(F), 5′-AGCCCCATAAGGTAGGCAGA-3′ (R); for *β*-MHC: 5′GCATTCTCCTGCTGTTTCCTT-3′ (F) and 5′-TGGATTCTCAAACGTGTCTAGTGA-3′ (R); for GAPDH: 5′-TGCCCCCATGTTTGTGATG-3′(F), 5′-TGTGGTCATGAGCCCTTCC-3′(R). The average Ct for each triplicate from qRT-PCR was used for analysis. Fold change in microRNA (or mRNA) expression was calculated by ΔΔCt, normalized with ΔCt = AvgCtmicroRNA − AvgCtU6 (or GAPDH).

### 2.6. Western-Blot

Total proteins of cell lysates and mitochondrial fractions were extracted by cell lysis buffer (50 mM Tris HCL (pH 7.4), 150 mM NaCL, 1 mM EDTA, 1% TRITON X-100), and equal amounts of protein from each lysate was separated by SDS-PAGE and transferred onto a nitrocellulose filter membrane (Millipore). The membrane was blocked and incubated at 4°C overnight with primary antibodies COX4 (Santa Cruz, USA) and GAPDH (EASYBIO), respectively. Then the membrane was washed and incubated at room temperature with secondary antibodies. Proteins were detected by ECL kit (CWBIO) and visualized on an imaging system (Bio-Rad).

### 2.7. Prediction of MicroRNA Targets and Pathway Enrichment Analysis

Target predictions of differentially expressed microRNAs were performed using the TarBase v7.0 database [21]. KEGG pathway (http://www.kegg.jp/kegg/pathway.html) enrichment analyses of the putative targets were performed using the mirPath v.3 software [22]. False discovery rate (FDR) < 0.05 and MicroT score > 0.9 were set as the thresholds for enrichment analyses. *p* values, hits of microRNAs, and hits of putative targets were summarized for each enriched pathway.

### 2.8. Statistical Analysis

Sequencing data was analysed by BGI (Beijing Genome Institute). Clean reads were searched for microRNA sequences using miRBase 21 to identify the known microRNAs in* Musculus*. The expression pattern of the sequencing data was visualized by hierarchical clustering. Differential expression analysis of the microRNA data was performed using the exact Poisson test, and the *p* values of multiple tests were adjusted by the false discovery rate (FDR < 0.05). Quantitative data besides the microRNA sequencing data were expressed as the mean ± standard error mean (SEM). The statistical analyses were performed using the GraphPad Prism 5.0. Differences between two groups were compared by Student's *t*-test or Wilcox rank sum test. Statistical significance was defined as *p* < 0.05.

## 3. Results

### 3.1. Pressure Overload-Induced Ischemic Heart Failure in Mice

**Figure 1:**
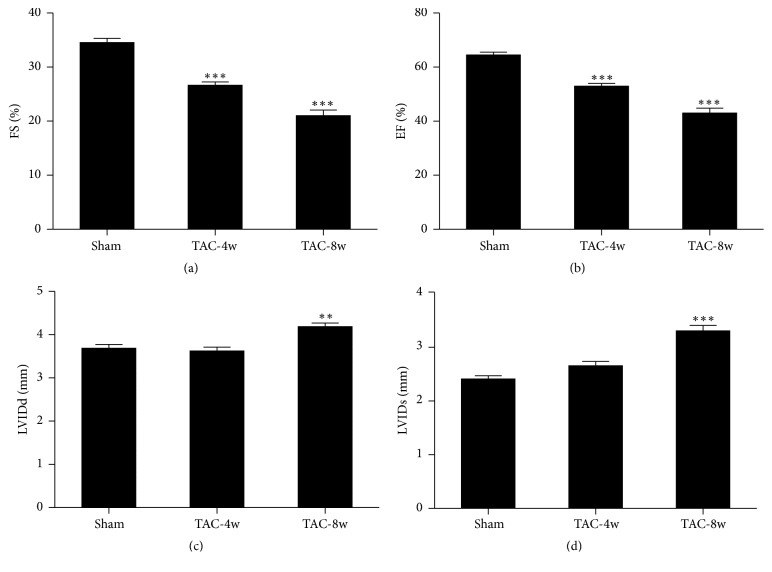
Functional characterization of hearts after TAC surgery measured by echocardiography. (a) Fractional shortening. (b) Ejection fraction. (c) Left ventricular end-diastolic internal diameter (LVIDd). (d) Left ventricular end-systolic internal diameter (LVIDs). *n* = 12 for each group. ^*∗∗*^*p* < 0.01, and ^*∗∗∗*^*p* < 0.001.

We generated a heart failure model in mice by a well-established transverse aortic constriction (TAC) surgery. Cardiac function was monitored by echocardiography (ECG) at 4 weeks (early stage) and 8 weeks (late stage) following TAC. The results showed that fractional shortening (FS) and ejection fraction (EF) were significantly reduced in TAC mice at both 4 weeks and 8 weeks (Figures 1(a) and 1(b)) compared with the sham control group. Meanwhile, left ventricular diastolic and systolic internal diameters (LVIDd and LVIDs) were increased only at 8 weeks (Figures 1(c) and 1(d)). These results indicated cardiac dysfunction in early and late stage of heart failure was induced by TAC.

### 3.2. Isolation and Purification of Heart Mitochondria

**Figure 2:**
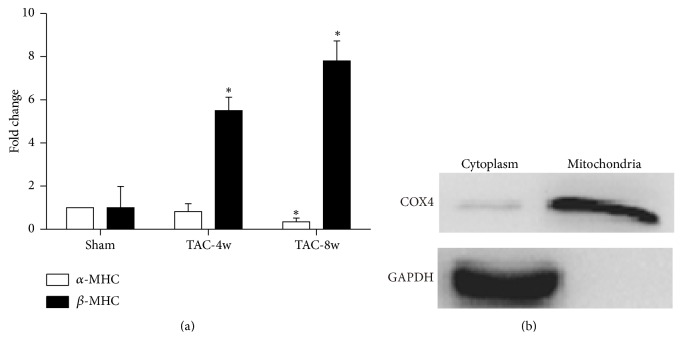
Measurement of heart failure marker expression and mitochondria purity. (a) Expression levels of *α*-MHC and *β*-MHC were quantified by real-time PCR. (b) Expression level of mitochondria-specific protein COX4 was determined by Western-blot; GAPDH was used as a control. ^*∗*^*p* < 0.05.

The gene expression shift from cardiac *α*-MHC to *β*-MHC has been considered as a molecular marker for heart failure. To confirm the occurrence of heart failure, we measured the expression levels of *α*-MHC and *β*-MHC by qPCR. *β*-MHC levels were markedly induced at both 4 weeks and 8 weeks following TAC, while *α*-MHC expression was decreased only at the end of 4 weeks (Figure 2(a)). This result was consistent with the cardiac function by ECG. Mitochondria were then isolated from the failing hearts and treated by RNase to remove RNAs on surface. To check quality and purity of the mitochondrial fractionation, we also measured expression of the mitochondrial gene COX4 by Western-blot. The results indicated mitochondria were successfully isolated (Figure 2(b)).

### 3.3. Mitochondria Associated MicroRNA Expression Profiling in Heart Failure

**Figure 3:**
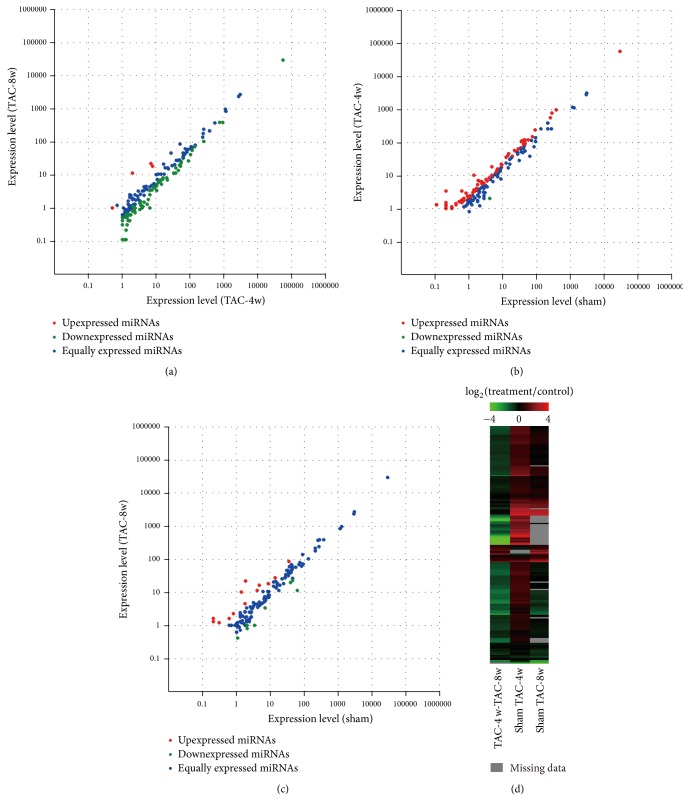
Comparisons of mitochondria associated microRNA expression profiling in heart failure at different stages. Scatter plots illustrate the microRNA expression levels between groups. (a) Comparison between TAC-8w and TAC-4w groups. (b) Comparison between TAC-4w and sham groups. (c) Comparison between TAC-8w and sham groups. (d) Heatmap representation for the differential expression of microRNAs.

To determine the expression profiling of mitochondria associated microRNA in heart failure, small RNA-seq was performed in pooled mitochondria RNA samples (*n* = 12) from different groups using Hiseq2500 Illumina. After preprocessing and filtering steps, we detected 362, 343, and 289 known microRNAs and 71, 47, and 43 predicted novel microRNAs in sham, TAC-4w, and TAC-8w heart tissues, respectively. The distribution of gene expression and comparison between different groups was demonstrated by Scatter plot. We found a wide upregulation of microRNA expression in 4 weeks' group compared to sham control, while microRNA levels in 8 weeks' group generally declined compared to those in 4 weeks (Figures 3(a)–3(c)). The consistent data were shown by clustered image map (Figure 3(d)), which included 193 paired microRNAs between sham and TAC-4w, 164 paired microRNAs between sham and TAC-8w, and 195 paired microRNAs between TAC-4w and TAC-8w groups. The differential expression of all the paired microRNAs was demonstrated in Supplementary Table S1 (see Supplementary Material available online at https://doi.org/10.1155/2017/4042509). We found 69 upregulated microRNAs (fold change > 2, *p* < 0.05) and only 2 microRNAs were downregulated (fold change < 0.5, *p* < 0.05) in TAC-4w group. In the TAC-8w group, 16 microRNAs were increased and 6 microRNAs were decreased compared with the sham control. The differentially expressed microRNAs between different groups were included in supplementary data (Supplementary Table) and the top differentially expressed microRNAs in TAC-4w and TAC-8w were shown in Table 1. Overall, the expression profiling of microRNAs associated with mitochondria showed a wide change in failing hearts.

### 3.4. Pathways Enriched by Differentially Expressed MicroRNAs from Mitochondria of Failing Hearts

**Figure 4:**
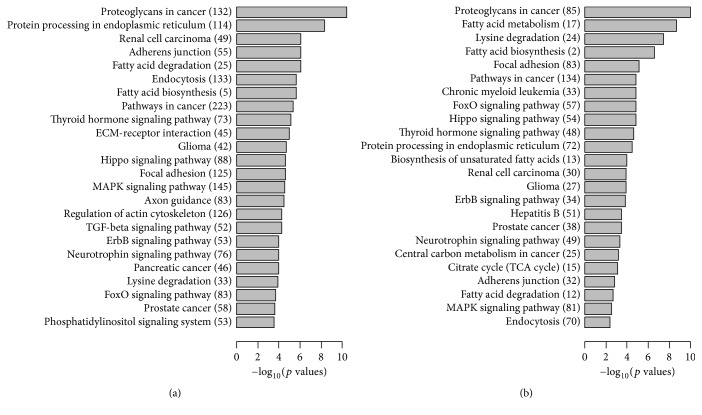
Target pathway enrichment of mitochondria associated microRNAs of failing hearts by KEGG pathway analysis. Numbers of microRNA targets are labeled in the bracket. (a) Enriched pathways in TAC-4w compared to sham were listed according to the *p* values. (b) Enriched pathways in TAC-8w compared to sham were listed according to the *p* values.

To find out the potential function of mitochondria associated microRNAs in heart failure, we performed KEGG pathway enrichment analysis for the putative targets of differentially expressed microRNAs. The complete lists of enriched pathways at early and late stage of HF were ranked according to the significance levels calculated by the miRpath software. The top enriched pathways at TAC-4w (Figure 4(a)) and TAC-8w (Figure 4(b)) were shown. We found that the most representative pathways relevant with fatty acid biosynthesis, metabolism, and degradation most frequently appeared in both groups. Moreover, thyroid hormone signaling, Hippo, and FOXO pathways, which are critical for energy metabolism regulation, were also present in both groups. These results suggested mitochondria associated microRNAs were involved in regulation of energy metabolism in failing heart. Additionally, the regulation of actin cytoskeleton and TGF-*β* signaling pathways was enriched in TAC-4w group, while citrate cycle regulatory pathway was found in TAC-8w group. The difference may reflect the regulation of reversible cardiac remodeling in the early stage and energy exhausting in the end stage of HF [23]. Taken together, the function of mitochondria associated microRNAs may play important roles in heart failure.

### 3.5. Identification of Mitochondria-Specific MicroRNAs by Quantitative PCR

**Figure 5:**
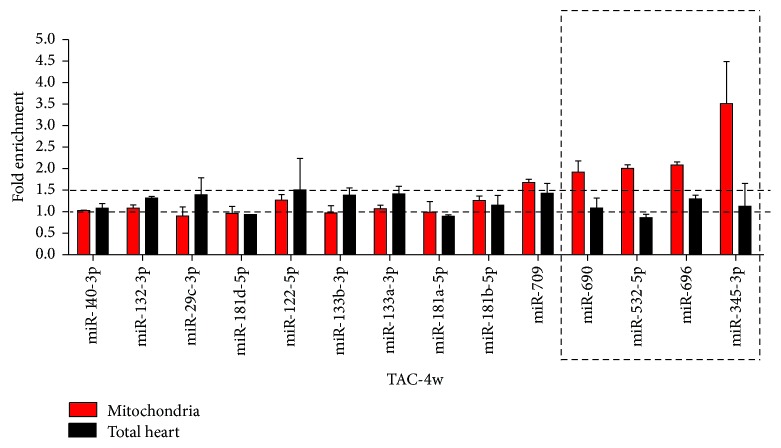
Identification of mitochondria enriched microRNAs of failing hearts (*n* = 12) by real-time PCR. Expression levels of mitochondrial microRNAs and total RNA from hearts were measured at 4 weeks after TAC.

Early changes in gene expression determined the development of HF and impaired mitochondrial function was considered an upstream signal to affect nonmitochondrial pathways in HF [24, 25]. We therefore considered mitochondria associated microRNAs in TAC-4w group, which showed generally increased levels, were critical candidates for regulating progress of HF. Therefore, 15 microRNAs in the group were chosen according to their expression changes in RNA-seq data. Real-time PCR was performed to validate their expression levels, the results were shown in Figure 5 (red column).

Differential expression of a large amount of cytoplasmic microRNAs were also reported in heart failure. Although the mitochondria were highly purified, contamination of cytosol components was hardly excluded. To identify microRNAs specifically enriched in mitochondria, we measured the levels of selected microRNAs in total RNAs from hearts (Figure 5, black column). By comparison with the expression levels in total RNAs, we found 4 mitochondrial microRNAs (mmu-miR-690, mmu-miR-532-5p, mmu-miR-696, and mmu-miR-345-3p) were enriched. Their expression increased in mitochondria but decreased or was unchanged in total RNAs, implying a mitochondria-specific translocation or regulation. The major functions of the 4 microRNAs are associated with mitochondria biogenesis and fission, fatty acids metabolism, oxidative stress, and apoptosis.

### 3.6. Identification of Putative MicroRNA Targets and Pathway Enrichment Analysis

**Figure 6:**
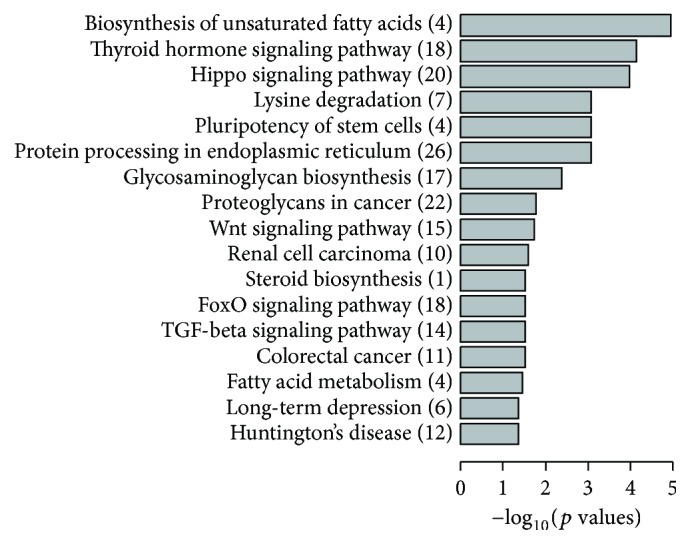
Target pathway enrichment of 4 mitochondria associated microRNAs by KEGG pathway analysis. Enriched pathways were listed according to their *p* values. Numbers of microRNA targets are labeled in the bracket.

We performed pathway enrichment analysis for the 4 microRNAs enriched in mitochondria. The TarBase dataset revealed a total of 755 putative targets for mmu-miR-532-5p, 157 for mmu-miR-690, 253 for mmu-miR696, and 11 for mmu-miR-345-3p. The most representative pathways involved in the heart failure were biosynthesis of unsaturated fatty acids, thyroid hormone signaling pathway, and hippo signaling pathway. The pathways were highly associated with energy metabolism (Figure 6).

## 4. Discussion

Mitochondria are considered as critical integrators of energy production, reactive oxygen species generation, and cell death pathways in the high energy-consuming beating heart. In this study, we for the first time measured microRNAs from mitochondria during the development of HF. It was found that mitochondria associated microRNAs were involved in energy metabolism pathways in the development of HF. The global profiling of microRNAs within the cytoplasm of cardiomyocytes during heart failure was examined in previous studies [26, 27]. However, no consistent “signature” microRNA profile was found common in these studies. MicroRNAs in HF play an extensive role in regulating hypotrophy, apoptosis, calcium handling, fibrosis, NF-kappa B, and TGF-*β* signaling pathways [4, 28, 29]. The current findings indicated a specific function of mitochondria associated microRNAs in heart failure. MicroRNA detection from mitochondria may be an effective way to monitor the development of heart failure.

Mitochondrial genome is only 16 kb long and it encodes 37 transcripts. Only a few microRNAs, that is, miR-181c, miR-4485, and miR-1973, were suggested to have a mitochondrial origin [14, 30, 31]. Most “mitomiRs” that have been detected in mitochondrial specimens are transcribed from nuclei and translocate to mitochondria. The microRNAs isolated from mitochondria include mitomiRs and inevitable contaminations from cytosol microRNAs, although mitochondria were treated by RNase. To identify mitochondria enriched microRNAs, we compared expression levels of microRNAs from mitochondria and total RNAs. Four microRNAs were found specifically enriched in mitochondria of failing heart. Their expression levels increased only in mitochondria but remained unchanged or decreased in total RNAs. These novel mitomiR candidates may be key regulators in heart failure.

We predicted several microRNA targeted pathways: fatty acids biosynthesis, thyroid hormone, and hippo signaling pathway enriched in mitochondria of HF. The pathways were associated with energy metabolism, which provided supporting evidences of their specific roles in regulating mitochondria. Previous studies suggested that the shift of metabolic substrate preference from fatty acid oxidation toward glucose oxidation was associated with the development of HF [32, 33]. A calcium induced increase in matrix volume led to stimulation of fatty acid oxidation in heart mitochondria [34]. Regulation of biosynthesis of unsaturated fatty acids by microRNAs is expected to alter the balance of energy metabolism and progression of cardiac hypertrophy. Thyroid hormone (TH) pathway regulates cardiac function through multiple mechanisms. TH is a key regulator of mitochondrial biogenesis and an inhibitor of mitochondrial oxidative stress. The pathway induced a rapid activation of metabolic pathways in heart and showed a direct role in transactivation of mitochondrial DNA [35]. Hippo pathway contributed to heart remodeling by regulating cell death during pressure overload initiated by TAC [36].

The major known function of miR-696 was to regulate the biogenesis of mitochondria and fatty acid oxidation in myocytes by targeting PGC-1*α* [37]. MiR-696 increased the lipid deposition and decreased the mitochondrial content in C2C12 cells [38] and regulated gluconeogenesis and insulin resistance in the liver from ob/ob mice [39]. As the predicted targets of mmu-miR-696, Irs1 is reported to cause heart failure in heart-specific IRS1/IRS2 double-knockout mice [40]. Our data showed the enrichment of miR-696 in mitochondria of the failing heart, which was consistent with the known role of miR-696 in mitochondria but suggested a novel function in heart failure. The predicted targets of miR-532-5p were Med1, NFATfatc1, and RBM20, which were reported to play crucial roles in the pathogenesis of heart failure [41–43]. The function of miR-690 in heart was still unknown. It was enriched in the nucleus of myeloid cells [44]. Its known function was related to metabolism and differentiation [45, 46]. MiR-690 was responsible for high glucose treatment in pancreatic beta cells [47]; it regulated Runx2-induced osteogenic differentiation [45]. Little was known about the function of miR-345-3p, yet several reports suggested the involvement of miR-345-3p in diabetic cardiomyopathy and the response to stress and oxidative stress [48, 49].

Nonmyocytes such as fibroblasts, endothelial cells, and immunocytes cells are also involved in the process of heart failure [50, 51]. The cell proportion varies during heart failure, which may result in differences of mitochondria microRNA levels. A limitation of this* in vivo* study is that mitochondria microRNAs of the nonmyocytes could hardly be excluded from those of cardiomyocytes. However, it is undoubted that cardiomyocytes are major responders in the remodeling process as specific functional cells, and ventricular cell hypertrophy is the morphological feature of the heart tissues at 4 weeks after TAC. Moreover, the number of the mitochondria increases in the development of cell hypertrophy. Hence, mitochondria in cardiomyocytes contribute a lot to the differentially expressed microRNAs in heart tissues. We reported here that the microRNAs were enriched in the mitochondria of failing heart and these mitochondria associated microRNAs may have potential roles in heart failure. Further studies will be performed to investigate their functions and mechanisms.

## 5. Conclusions

In summary, the current study has provided the first evidence that mitochondria associated microRNAs may contribute to the regulation of energy metabolism pathways. We performed differential microRNA expression profile analysis for mitochondria associated microRNAs during development of heart failure and highlighted pathways regulated by these microRNAs. The methods used in this study were proved to be an effective way to understand microRNA-mitochondria interactions and to identify potential targets for diagnosis and treatment of heart failure.

## Supplementary Material

Expression changes of microRNAs by RNA-seq.

## Figures and Tables

**Table 1:** List of the top differentially expressed microRNAs from heart mitochondria in TAC mice.

Pairwise	Upregulated microRNAs			Downregulated microRNAs		
	miR-name	Fold change (log2 TAC/Sham)	*p* value	miR-name	Fold change (log2 TAC/Sham)	*p* value
Sham- 4-week _TAC	mmu-miR-345-3p	6.67582	0.015490	mmu-miR-145a-3p	−1.06684	0.00531
	mmu-miR-532-5p	4.09083	9.83*E* − 09	mmu-miR-200b-3p	−2.9964	0.021703
	mmu-miR-709	3.70383	0.000961			
	mmu-miR-181b-5p	3.70383	0.000961			
	mmu-miR-133a-3p	3.17332	0.011587			
	mmu-miR-690	3.00339	0.021268			
	mmu-miR-133b-3p	3.00339	0.021268			
	mmu-miR-696	2.91028	0.001289			
	mmu-miR-122-5p	2.86861	5.72*E* − 18			
	mmu-miR-181a-5p	2.81075	0.038716			
	mmu-miR-140-3p	2.70383	0.004118			

Sham- 8-week _TAC	mmu-miR-6538	3.54852	5.29*E* − 45	mmu-miR-34c-5p	−1.04088	0.000366
	mmu-miR-299a-5p	3.21129	0.010257	mmu-miR-696	−1.35071	0.019921
	mmu-miR-199a-5p	3.04141	0.019076	mmu-miR-345-3p	−1.72393	0.000356
	mmu-miR-5126	3.04141	0.019076	mmu-miR-132-3p	−2.47141	3.24*E* − 88
	mmu-miR-532-5p	3.04136	0.000584	mmu-miR-181a-2-3p	−2.95879	0.024148
	mmu-miR-148a-3p	2.87806	5.57*E* − 18	mmu-miR-200b-3p	−7.08725	0.004443
	mmu-miR-208a-3p	2.74182	0.003498			
	mmu-miR-7225-5p	2.04169	0.01827			
	mmu-miR-133b-3p	1.82293	3.62*E* − 17			
	mmu-miR-28a-3p	1.62637	0.16591			
	mmu-miR-146a-5p	1.50101	0.008471			
	mmu-miR-709	1.48215	1.64*E* − 09			

## References

[1] Gustafsson A. B., Gottlieb R. A. (2008). Heart mitochondria: gates of life and death. *Cardiovascular Research*.

[2] Bayeva M., Gheorghiade M., Ardehali H. (2013). Mitochondria as a therapeutic target in heart failure. *Journal of the American College of Cardiology*.

[3] Bartel D. P. (2004). MicroRNAs: genomics, biogenesis, mechanism, and function. *Cell*.

[4] Cheng Y., Ji R., Yue J. (2007). MicroRNAs are aberrantly expressed in hypertrophic heart: do they play a pole in cardiac hypertrophy?. *The American Journal of Pathology*.

[5] Greco S., Fasanaro P., Castelvecchio S. (2012). MicroRNA dysregulation in diabetic ischemic heart failure patients. *Diabetes*.

[6] Montgomery R. L., Hullinger T. G., Semus H. M. (2011). Therapeutic inhibition of miR-208a improves cardiac function and survival during heart failure. *Circulation*.

[7] Su M., Wang J., Wang C. (2015). MicroRNA-221 inhibits autophagy and promotes heart failure by modulating the p27/CDK2/mTOR axis. *Cell Death and Differentiation*.

[8] Zhou J., Gao J., Zhang X. (2015). microRNA-340-5p functions downstream of cardiotrophin-1 to regulate cardiac eccentric hypertrophy and heart failure via target gene dystrophin. *International Heart Journal*.

[9] Li H., Fan J., Yin Z., Wang F., Chen C., Wang D. W. (2016). Identification of cardiac-related circulating microRNA profile in human chronic heart failure. *Oncotarget*.

[10] Hwang H.-W., Wentzel E. A., Mendell J. T. (2007). A hexanucleotide element directs microRNA nuclear import. *Science*.

[11] Bandiera S., Rüberg S., Girard M. (2011). Nuclear outsourcing of RNA interference components to human mitochondria. *PLoS ONE*.

[12] Geiger J., Dalgaard L. T. (2017). Interplay of mitochondrial metabolism and microRNAs. *Cellular and Molecular Life Sciences*.

[13] Zhang X., Zuo X., Yang B. (2014). MicroRNA directly enhances mitochondrial translation during muscle differentiation. *Cell*.

[14] Das S., Ferlito M., Kent O. A. (2012). Nuclear miRNA regulates the mitochondrial genome in the heart. *Circulation Research*.

[15] Carrer M., Liu N., Grueter C. E. (2012). Control of mitochondrial metabolism and systemic energy homeostasis by microRNAs 378 and 378^∗^. *Proceedings of the National Academy of Sciences of the United States of America*.

[16] Wang H., Li J., Chi H. (2015). MicroRNA-181c targets Bcl-2 and regulates mitochondrial morphology in myocardial cells. *Journal of Cellular and Molecular Medicine*.

[17] Bandiera S., Matégot R., Girard M., Demongeot J., Henrion-Caude A. (2013). MitomiRs delineating the intracellular localization of microRNAs at mitochondria. *Free Radical Biology and Medicine*.

[18] Srinivasan H., Das S. (2015). Mitochondrial miRNA (MitomiR): A new player in cardiovascular health. *Canadian Journal of Physiology and Pharmacology*.

[19] Jagannathan R., Thapa D., Nichols C. E. (2015). Translational Regulation of the Mitochondrial Genome Following Redistribution of Mitochondrial MicroRNA in the Diabetic Heart. *Circulation: Cardiovascular Genetics*.

[20] Kumarapeli A. R. K., Su H., Huang W. (2008). *α*b-crystallin suppresses pressure overload cardiac hypertrophy. *Circulation Research*.

[21] Vlachos I. S., Paraskevopoulou M. D., Karagkouni D. (2015). DIANA-TarBase v7.0: indexing more than half a million experimentally supported miRNA:mRNA interactions. *Nucleic Acids Research*.

[22] Vlachos I. S., Zagganas K., Paraskevopoulou M. D. (2015). DIANA-miRPath v3.0: deciphering microRNA function with experimental support. *Nucleic Acids Research*.

[23] Hein S., Kostin S., Heling A., Maeno Y., Schaper J. (2000). The role of the cytoskeleton in heart failure. *Cardiovascular Research*.

[24] Buermans H. P. J., Redout E. M., Schiel A. E. (2005). Microarray analysis reveals pivotal divergent mRNA expression profiles early in the development of either compensated ventricular hypertrophy or heart failure. *Physiological Genomics*.

[25] Dai D.-F., Hsieh E. J., Chen T. (2013). Global proteomics and pathway analysis of pressure-overload-induced heart failure and its attenuation by mitochondrial-targeted peptides. *Circulation: Heart Failure*.

[26] Bagnall R. D., Tsoutsman T., Shephard R. E., Ritchie W., Semsarian C. (2012). Global microRNA profiling of the mouse ventricles during development of severe hypertrophic cardiomyopathy and heart failure. *PLoS ONE*.

[27] Zhu X., Wang H., Liu F. (2013). Identification of micro-RNA networks in end-stage heart failure because of dilated cardiomyopathy. *Journal of Cellular and Molecular Medicine*.

[28] Prasad S. V. N., Gupta M. K., Duan Z.-H. (2017). A unique microRNA profile in end-stage heart failure indicates alterations in specific cardiovascular signaling networks. *PLoS ONE*.

[29] Liu X., Meng H., Jiang C., Yang S., Cui F., Yang P. (2016). Differential microRNA expression and regulation in the rat model of post-infarction heart failure. *PLoS ONE*.

[30] Bianchessi V., Badi I., Bertolotti M. (2015). The mitochondrial lncRNA ASncmtRNA-2 is induced in aging and replicative senescence in Endothelial Cells. *Journal of Molecular and Cellular Cardiology*.

[31] Sripada L., Singh K., Lipatova A. V. (2017). hsa-miR-4485 regulates mitochondrial functions and inhibits the tumorigenicity of breast cancer cells. *Journal of Molecular Medicine*.

[32] Lemieux H., Bulteau A. L., Friguet B., Tardif J.-C., Blier P. U. (2011). Dietary fatty acids and oxidative stress in the heart mitochondria. *Mitochondrion*.

[33] Den Ruijter H. M., Verkerk A. O., Schumacher C. A. (2012). A diet rich in unsaturated fatty acids prevents progression toward heart failure in a rabbit model of pressure and volume overload. *Circulation: Heart Failure*.

[34] Halestrap A. P. (1987). The regulation of the oxidation of fatty acids and other substrates in rat heart mitochondria by changes in the matrix volume induced by osmotic strength, valinomycin and Ca^2+^. *The Biochemical Journal*.

[35] Forini F., Nicolini G., Iervasi G. (2015). Mitochondria as key targets of cardioprotection in cardiac ischemic disease: Role of thyroid hormone triiodothyronine. *International Journal of Molecular Sciences*.

[36] Zhang Y., Del Re D. P. (2017). A growing role for the Hippo signaling pathway in the heart. *Journal of Molecular Medicine*.

[37] Aoi W., Naito Y., Mizushima K. (2010). The microRNA miR-696 regulates PGC-1*α* in mouse skeletal muscle in response to physical activity. *The American Journal of Physiology—Endocrinology and Metabolism*.

[38] Wen F., Zhang H., Bao C. (2015). Resistin Increases Ectopic Deposition of Lipids Through miR-696 in C2C12 Cells. *Biochemical Genetics*.

[39] Fang Z., Li P., Jia W., Jiang T., Wang Z., Xiang Y. (2016). miR-696 plays a role in hepatic gluconeogenesis in ob/ob mice by targeting PGC-1*α*. *International Journal of Molecular Medicine*.

[40] Qi Y., Xu Z., Zhu Q. (2013). Myocardial loss of IRS1 and IRS2 causes heart failure and is controlled by p38*α* MAPK during insulin resistance. *Diabetes*.

[41] Spitler K. M., Ponce J. M., Oudit G. Y., Hall D. D., Grueter C. E. (2017). Cardiac med1 deletion promotes early lethality, cardiac remodeling, and transcriptional reprogramming. *American Journal of Physiology - Heart and Circulatory Physiology*.

[42] Phoon C. K. L., Ji R. P., Aristizábal O. (2004). Embryonic heart failure in NFATc1-/- mice novel: Mechanistic insights from in utero ultrasound biomicroscopy. *Circulation Research*.

[43] Guo W., Schafer S., Greaser M. L. (2012). RBM20, a gene for hereditary cardiomyopathy, regulates titin splicing. *Nature Medicine*.

[44] Wong J. J. L., Ritchie W., Gao D. (2014). Identification of nuclear-enriched miRNAs during mouse granulopoiesis. *Journal of Hematology and Oncology*.

[45] Yu S., Geng Q., Pan Q. (2016). MiR-690, a Runx2-targeted miRNA, regulates osteogenic differentiation of C2C12 myogenic progenitor cells by targeting NF-kappaB p65. *Cell and Bioscience*.

[46] Chang L., Qi H., Xiao Y. (2016). Integrated analysis of noncoding RNAs and mRNAs reveals their potential roles in the biological activities of the growth hormone receptor. *Growth Hormone and IGF Research*.

[47] Tang X., Muniappan L., Tang G., Özcan S. (2009). Identification of glucose-regulated miRNAs from pancreatic *β* cells reveals a role for miR-30d in insulin transcription. *RNA*.

[48] Chavali V., Tyagi S. C., Mishra P. K. (2014). Differential expression of dicer, miRNAs, and inflammatory markers in diabetic Ins2+/- Akita hearts. *Cell Biochemistry and Biophysics*.

[49] Zheng Y., Chen K.-L., Zheng X.-M., Li H.-X., Wang G.-L. (2014). Identification and bioinformatics analysis of microRNAs associated with stress and immune response in serum of heat-stressed and normal Holstein cows. *Cell Stress and Chaperones*.

[50] Tijsen A. J., Pinto Y. M., Creemers E. E. (2012). Non-cardiomyocyte microRNAs in heart failure. *Cardiovascular Research*.

[51] Yamagami K., Oka T., Wang Q. (2015). Pirfenidone exhibits cardioprotective effects by regulating myocardial fibrosis and vascular permeability in pressure-overloaded hearts. *American Journal of Physiology - Heart and Circulatory Physiology*.

